# Supplementary material for: Short-term reward experience biases inference despite dissociable neural correlates
Source: Nat Commun. 2017 Nov 22;8:1690. doi: 10.1038/s41467-017-01703-0 (PMC5700163; doi:10.1038/s41467-017-01703-0)
Supplement: Supplementary file 1 — Supplementary Information [file 41467_2017_1703_MOESM1_ESM.pdf]

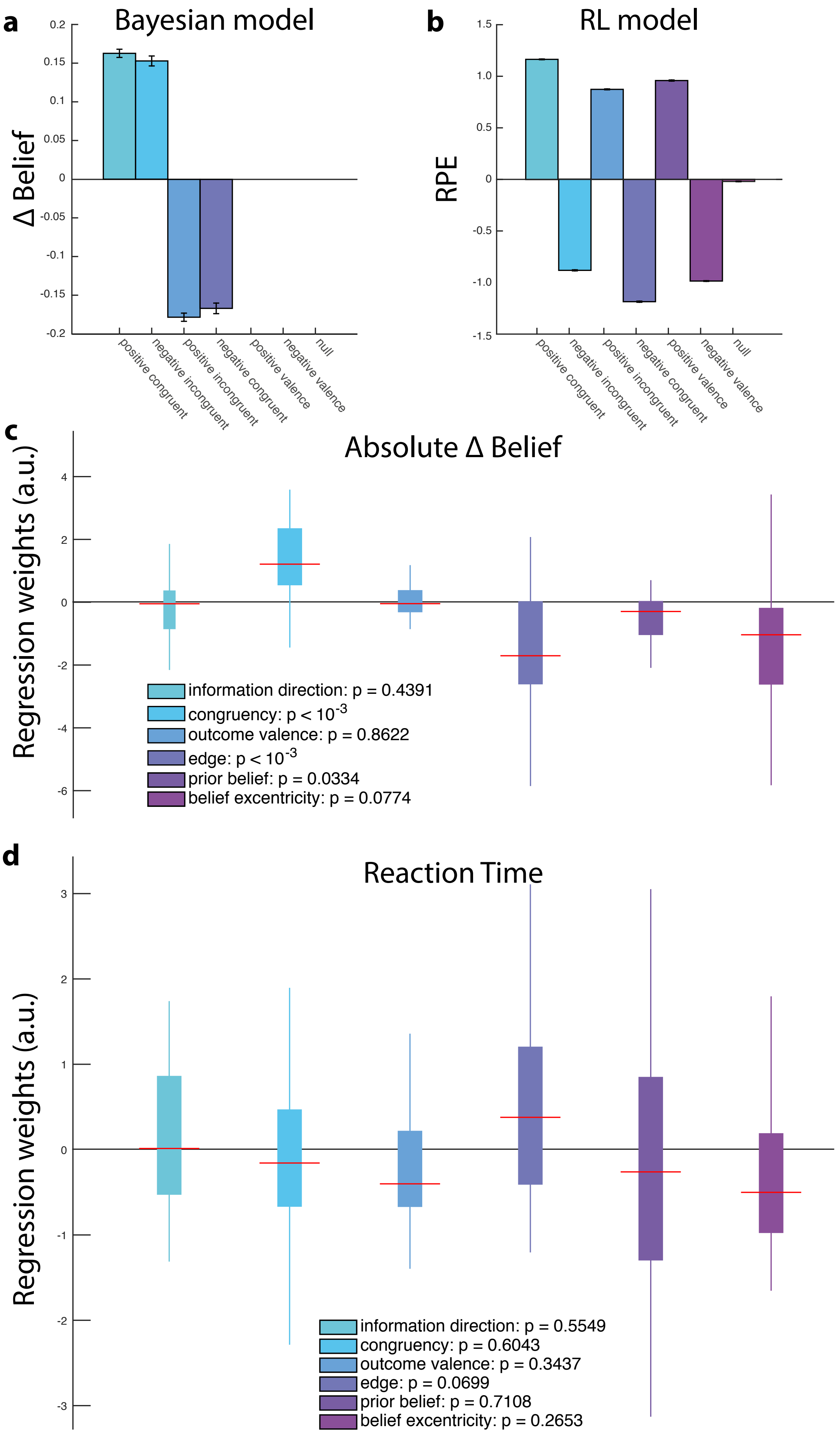

a
Bayesian model
Δ Belief
0.2
0.15
0.1
0.05
0
-0.05
-0.1
-0.15
-0.2
positive congruent
negative incongruent
positive incongruent
negative congruent
positive valence
negative valence
null
b
RL model
RPE
1.5
1.0
0.5
0
-0.5
-1.0
-1.5
positive congruent
negative incongruent
positive incongruent
negative congruent
positive valence
negative valence
null
c
Absolute Δ Belief
Regression weights (a.u.)
4
2
0
-2
-4
-6
information direction: p = 0.4391
congruency: p < 10-3
outcome valence: p = 0.8622
edge: p < 10-3
prior belief: p = 0.0334
belief excentricity: p = 0.0774
d
Reaction Time
Regression weights (a.u.)
3
2
1
0
-1
-2
-3
information direction: p = 0.5549
congruency: p = 0.6043
outcome valence: p = 0.3437
edge: p = 0.0699
prior belief: p = 0.7108
belief excentricity: p = 0.2653

Supplementary Figure 1 | Modelled human behaviour, belief updating and reaction times at belief prompt.

Given the exact sequence of trials that each human participant chose in the task, model predictions were slightly different due to the effect of imbalanced choices between good and bad urns. The Bayesian model (**a**) updated slightly more on negative congruent and positive incongruent events. This is likely caused by participants choosing good urns more often than bad urns, which leads to higher degrees of certainty for those events that occur more often in good urns, resulting in less update. The (normalized) prediction error (**b**) closely follows outcome magnitudes. Note that given the larger absolute prediction errors on congruent outcomes, the RL model over time converges to the correct value. The very narrow error-bars here are the result of re-scaling outcomes to the respective highest possible outcome to account for range adaptation effects[1,2]. The slightly negative RPE for null events is explained by the expected value being above 0 an average.

Results of a regression analysis of relevant task factors against absolute belief updating (**c**) and reaction times (RT) until the belief prompt was confirmed after each trial (**d**). There was a significant (t_{23} = 4.06, p = 0.00049) positive effect of factor *congruency* on belief updating, yet not RT (t_{23} = -0.52, p = 0.60), confirming that participants updated their beliefs less when model-free experience and model-based inference were incongruent with each other. Neither the direction of the belief update (increase or decrease), nor the valence (positive or negative) itself had an effect on belief updating or RTs. Factor *edge* accounts for possible edge effects, in that participants update less (t_{23} = -3.82, p = 0.00087) when they are certain about the property of an urn, which is seen on-top of a trend (t_{23} = -1.87, p = 0.0774) of *belief excentricity* (the absolute deviation from a flat prior), which both suggest Bayesian belief updating. The *prior belief* in an urn to be good is also associated with reduced updating (t_{23} = -2.26, p = 0.0334). This effect is likely caused by more choices of good compared to bad urns, which thus are updated less, or, vice versa, higher updating on possibly more exploratory choices of bad urns.

The absence of effects on RTs is likely explained by the delay between feedback presentation and onset of the belief prompt, which appears to have provided sufficient time to process both congruent and incongruent information. Note that inclusion of a separate regressor coding the delay between feedback and belief prompt did neither show a main effect (p > 0.78) or interaction with *congruency* (p > 0.71), nor alter any of the other non-significant findings.

Error-bars reflect SE and statistical values are results of separate *t*-tests of standardized regression weights of within-participants effects against zero. In the whisker plots, red horizontal line = median, box = quartile range, whiskers = range.

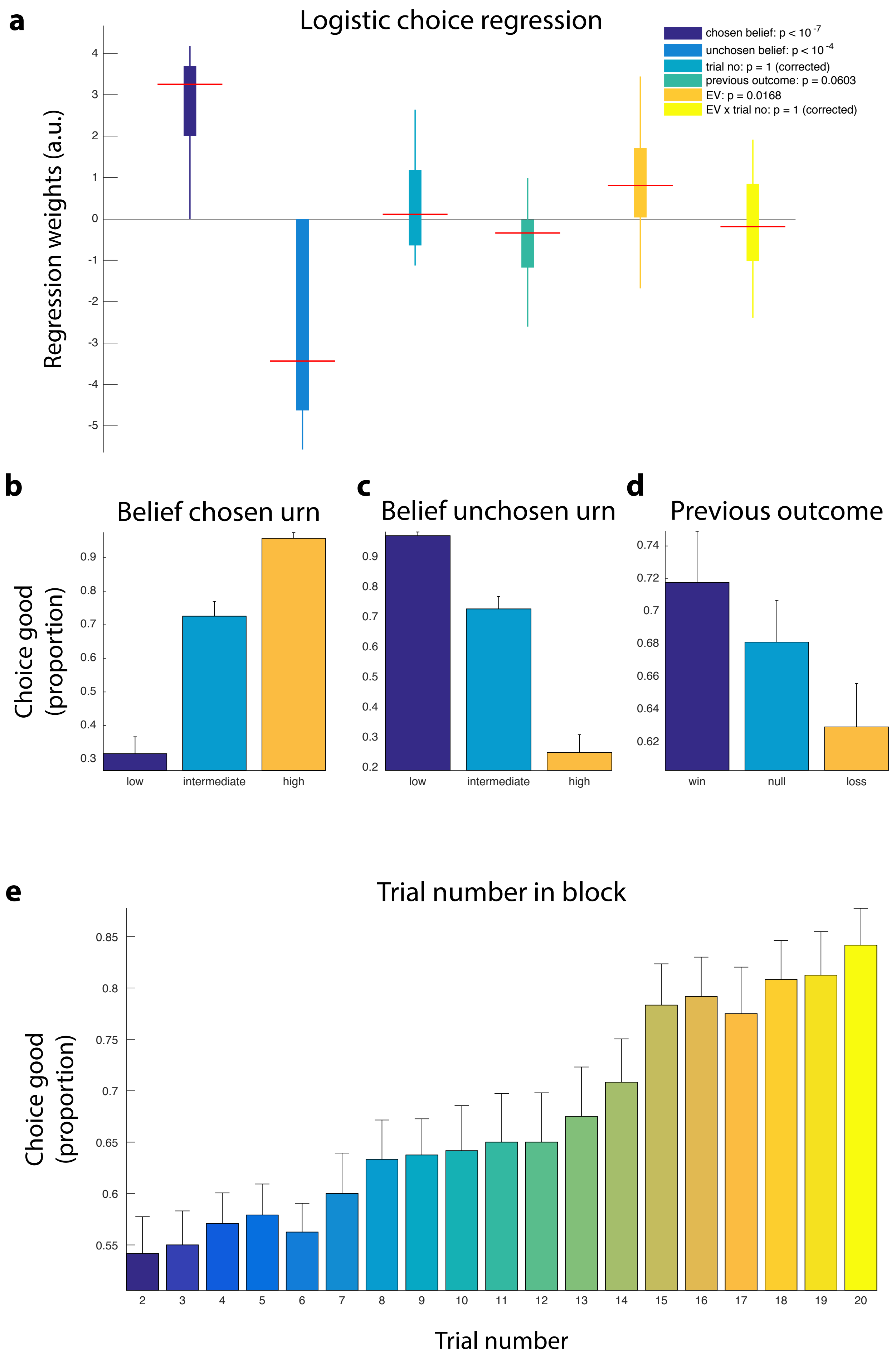

a
Logistic choice regression
chosen belief: p < 10 -7
unchosen belief: p < 10 -4
trial no: p = 1 (corrected)
previous outcome: p = 0.0603
EV: p = 0.0168
EV x trial no: p = 1 (corrected)
Regression weights (a.u.)
4
3
2
1
0
-1
-2
-3
-4
-5
b
Belief chosen urn
Choice good (proportion)
0.9
0.8
0.7
0.6
0.5
0.4
0.3
low
intermediate
high
c
Belief unchosen urn
0.9
0.8
0.7
0.6
0.5
0.4
0.3
0.2
low
intermediate
high
d
Previous outcome
0.74
0.72
0.7
0.68
0.66
0.64
0.62
win
null
loss
e
Trial number in block
Choice good (proportion)
0.85
0.8
0.75
0.7
0.65
0.6
0.55
2
3
4
5
6
7
8
9
10
11
12
13
14
15
16
17
18
19
20
Trial number

Supplementary Figure 2 | Relationship between beliefs and choices.

To investigate the relationship between participants' beliefs and their choices, we conducted a logistic-regression onto participants' choices of urns when one was good and the other bad. We defined choices of good urns as 1 and of bad urns as 0. As regressors we included the valence of the previous outcome (-1 = loss of points, 0 = no change, 1 = gain of points), and the current trial number in the block (*trial no*) to account for general learning over time. Additionally, we included the relative expected value (*EV*) of the good or bad urn as if participants had knowledge of this, as well as the interaction *expected value* x *trial no*, to test if the participants might have counted events that had not been drawn. The relative *EV* depends on how many incongruent compared to congruent events are left. For example, had a participant counted all received events, and in the last trial only an incongruent marble was left, the better choice would be to select a bad and not a good urn. We first test the influence these factors have on participants' choices in a logistic regression model, and then compare this model to another one that includes the belief of the chosen and the belief of the unchosen urn to be good. Furthermore, we plot raw quantile splits of the data, which show the effect of the included factors separately, that is, when variance by other factors is unaccounted for (**b-e**).

First, we find that inclusion of participants' beliefs dramatically increases model fit (r^2 = 0.177 without and r^2 = 0.704 with beliefs). Despite anticorrelation between the beliefs of chosen and unchosen urn (mean r = -0.49 ± 0.06), we found that the strongest factor driving participants' choices in the task, is the belief of an urn to be good (t_{23} = 9.25, p = $1.96\text{x}10^{-8}$) over and above the effect of believing the other urn to be bad (t_{23} = -6.31, p = 0.000016). This confirms the relevance of beliefs for choice behaviour. Furthermore, because shared variance in regression analysis is attributed to the error term, this indicates that participants employed information about the alternative urn, and chose a good urn more often when the alternative was estimated to be poor, as is also confirmed by the raw data splits (**b**,**c**).

In the regression without beliefs, in accordance with the raw data plots (**d**,**e**), we found a significant positive effect of trial number on the proportion of good urn choices (t_{23} = 5.97, p = $1.71\text{x}10^{-5}$) and a negative effect of the valence of the previous outcome (t_{23} = -4.36, p = 0.0009). Both effects were fully explained by beliefs and no longer significant in the regression including participants' beliefs (both p > 0.06). This indicates that beliefs in the current task mediate effects of learning over blocks and previous trial outcome effects onto choices, further confirming the behavioural relevance of beliefs and inference in the current task.

There was a significant effect of the expected value left on choices of the better urn (t_{23} = 3.35, p = 0.017). This is most likely because the expected value depends on how many congruent events have been observed, and more congruent events lead to more choices of good urns as these are unaffected by bias. However, there seems to be an additional effect of this on choices that is over and above the effect on belief, possibly indicating that choices are even more biased then inference. However, there is no interaction between expected value and trial number (t_{23} = -0.38, p = 0.71 uncorrected), indicating that participants did not count task events.

In (**a**) red horizontal line = median, box = quartile range, whiskers = range, error-bars in (**b-e**) = SE, p-values were corrected for the number of factors in the respective models by applying Bonferroni correction.

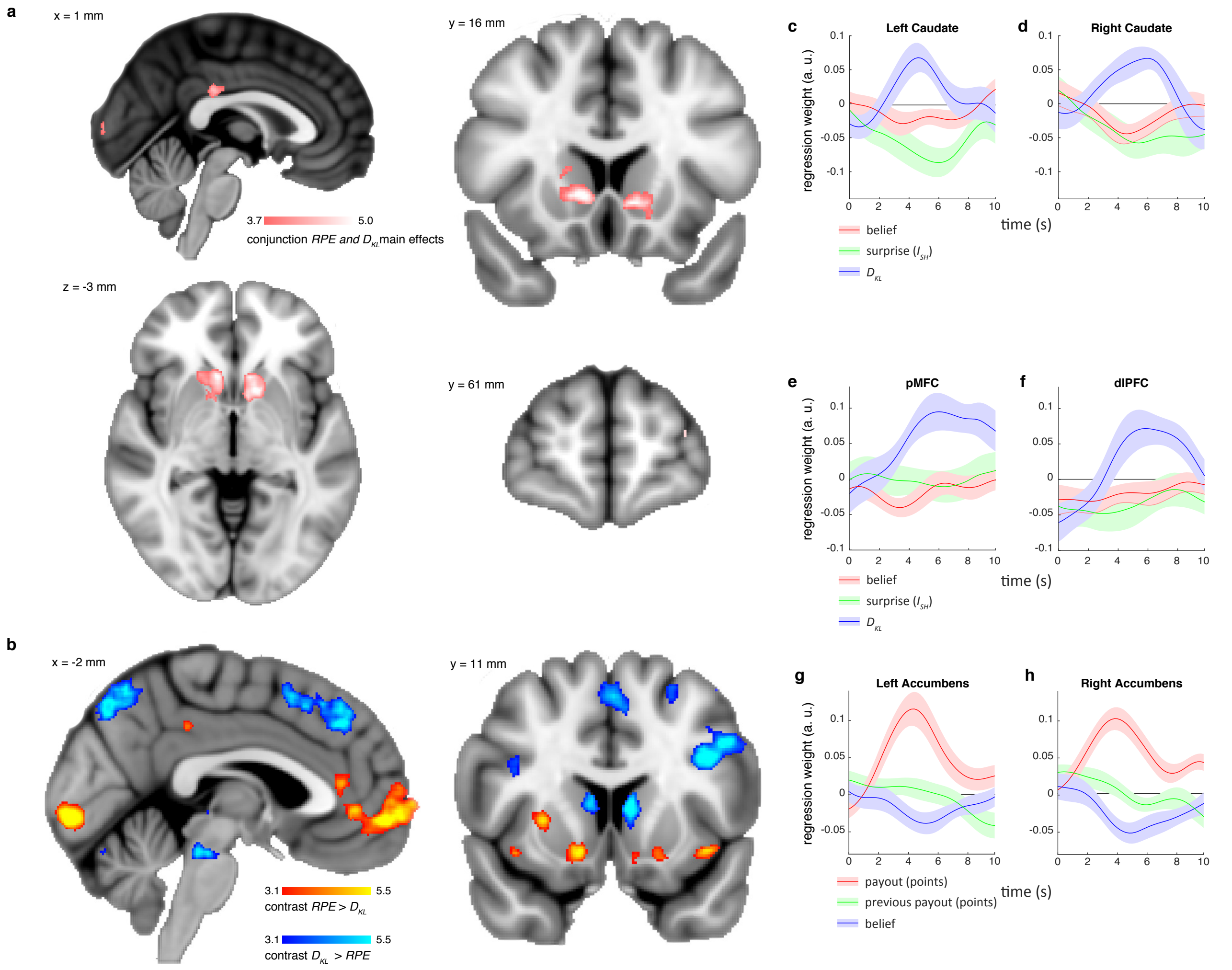

Supplementary Figure 3 | Conjunction effects and contrasts of D_{KL} and *RPE*, and signal characteristics in peak voxels of main effects.

(**a**) BOLD signals in middle striatum as well as lateral frontopolar and posteromedial cortex were jointly and significantly modulated by model-based (D_{KL}) and model-free learning (*RPE*).

(**b**) Contrasts for $D_{KL} > RPE$ (blue) and $D_{KL} < RPE$ (red) confirmed that effects in IPS (peak -13, -71, 54 mm, $z = 5.04$), left dlPFC (-45, 6, 34 mm, $z = 5.24$), pMFC (-4, 36, 40 mm, $z = 4.41$), and left dorsal striatum (-8, -15, 5 mm, $z = 4.34$) for D_{KL} were seen over the effect of *RPE*. Vice versa, bilateral ventral striatum (right 13, 12, -10 mm, $z = 4.07$; left -10, 12, -11 mm, $z = 3.21$) and vmPFC (-4, 54, -6 mm, $z = 5.30$) showed significantly larger covariation with *RPE* compared to D_{KL}. The contrasts for $D_{KL} > RPE$ in right dorsal striatum (8, 12, 10 mm, $z = 3.96$) did not survive cluster based FWE correction and the contrast in the left frontal pole, were a main effect was seen for D_{KL}, was not significant.

(**a**) displays minimum *z*-statistics of the conjunction null hypothesis of either factor showing no effect thresholded as in main Fig. 3**a**, and (**b**) displays *z*-statistics of both contrasts uncorrected for display purpose. Colourbars indicate *z*-scores.

(**c-h**) We further explored the results of the whole brain analysis by conducting several control analyses at the respective peak voxel of each main effect (striatum: D_{KL} left caudate -9, 16 , 1 mm, right caudate 9, 16, 6; RPE_t left

nucleus accumbens -13, 10, -9 mm, right nucleus accumbens 13, 10, -8 mm). A current discussion centres around the question whether some neural signals thought to reflect valenced outcome evaluation, such as reward prediction errors enabling learning, can actually be better explained by unsigned outcomes or surprise, for which signals in the pMFC and also the midbrain's dopaminergic system have been found[3-5]. Formally, surprise can be defined according to information theory as the Shannon Information of an event ($I_{SH}(E)$) as:

$$I_{SH}(E) = -log[p(E|prior)]$$

In our task, the prior belief is explicitly prompted which allows to directly compute I_{SH} from the probability distribution of the events provided to all participants. Note that all non-informative events always had the lowest possible probability of occurrence (0.1), which thus was independent of the prior belief. This leads to an anti-correlation of surprise and D_{KL} in our task (average r = -0.64 across participants, range: -0.938 to -0.37). Thus, an interpretation of the *positive* effect for D_{KL} based on *negative* surprise signals appears unlikely. However, we also investigated the effects of surprise and D_{KL} in multiple regression analyses within voxels of peak activities. Therefore, we included I_{SH} into a regression model as a separate factor in order to see if the effect of D_{KL} remained significant. (**c-f**) show regression time courses in peak voxels of main effects using a general linear model with the following predictors: D_{KL} (blue), I_{SH} (green), *belief* (that chosen urn is good, red), *current* and *previous payout*, *onset of belief prompt*, and *block number* (to account for different magnitudes in payout over blocks). In the dorsal striatum (**c, d**), we found a negative going effect of I_{SH} (peak left p = 0.0003, right p = 0.016) and the effect of D_{KL} remained significant despite the considerable amount of collinearity between both factors in some participants (peak left p = 0.0027, right p = 0.0003). Additionally, the D_{KL} main effect remained significant in all other regions reported in the main effects analysis when I_{SH} was included as a separate factor: (**e**) pMFC (-1, 28, 50 mm, p = 0.0003), (**f**) dlPFC (-45, 27, 27 mm, p = 0.0017), and IPS (-14, -70, 53 mm, p = 5.05×10^{-7}). This demonstrates that the neural correlates of long-term belief updating identified in the task cannot be reduced to surprise.

(**g, h**) Furthermore, to dissociate the constituent components of the reward prediction-error (outcome and expectancy), we also included the outcome as well as its expectancy into the regression model (instead of RPE_t). A true prediction error signal would require positive covariation with the outcome and negative covariation with its expectancy[6], yet the source of the expectancy could be speculated to vary. Because in this task maximum likelihood estimates for the learning rate of the RL model in various participants showed very low values (reflecting the difficulty to learn the task for an RL algorithm and the need for integration of outcomes over very long times), we tested the following hypotheses: we included the outcome of the preceding trial (green) as a separate regressor, reflecting immediate reward expectancy (or expected value in an RL model with a ceiling learning rate), as well as the actual belief (blue) entered on the previous trial for that urn (that is, a participant's expectancy of an urn to be good or bad). As demonstrated before, the

belief of the participants was strongly influenced by model-based learning (main Fig. 2) and, therefore, this analysis tests the hypothesis that model-based values can form the trial-by-trial prediction (expected value) for reward-prediction error correlates in the ventral striatum under some circumstances. We found strong positive covariation with the payout of the current trial (red) in the ventral striatum (**c, d**; peak left $p = 1.55 \times 10^{-5}$, right $p = 5.45 \times 10^{-7}$). However, the previous outcome did not systematically modulate the BOLD signal in the ventral striatum (and the same was found when we used two other, arbitrarily chosen learning rates of alpha = 0.06 (average of all participants), or 0.15, data not shown). However, we found significant (peak p = 0.0134 left and p = 0.001 right) covariation with the belief entered by the participant at the previous trial the same urn was chosen before. This indicates that even in the ventral striatum, value estimates do not merely reflect model-free evaluation, but include model-based inferences, similar to the finding of Daw et al.[7]. Note that this effect was observed over-and-above surprise, which was again also included in the model. Finally, the current belief otherwise was only found to exert a similar effect within the right caudate (**d**, p = 0.0038), yet not in other regions found in the D_{KL} main analysis.

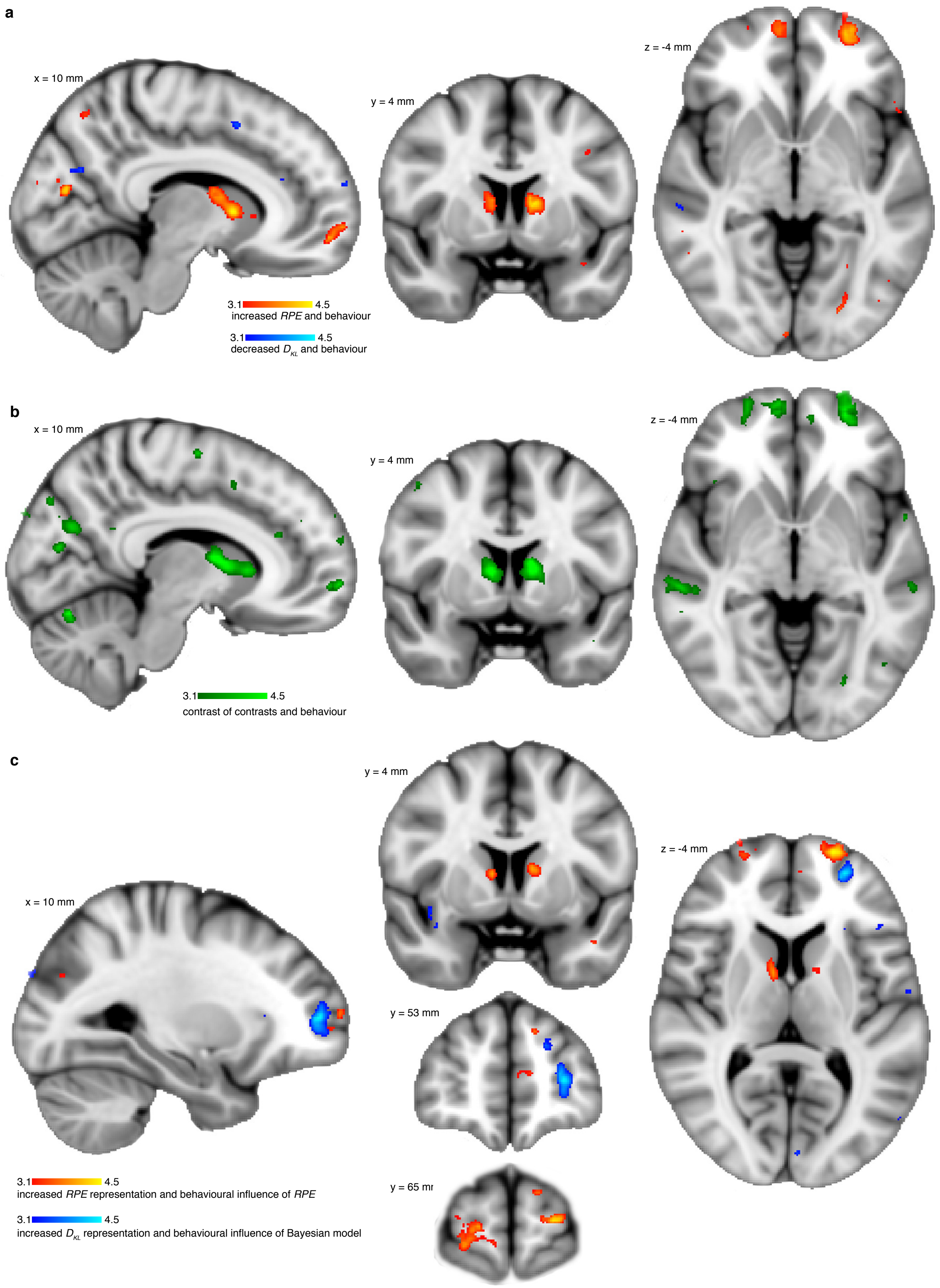

Supplementary Figure 4 | Increased *RPE* and not decreased D_{KL} representation drives the across-participants effects.

(a) When we regressed the behavioural influence of Bayesian and RL model update onto the coefficient parameter estimates for D_{KL} (blue) and *RPE* (red) separately, instead of using their contrast as in main Fig. 4, we found that the effect seen on the contrast of contrasts was driven mainly by stronger representation of *RPEs* in dorsal striatum as well as frontopolar cortex, instead of a reduction of Bayesian model information. Peak activity was found in right caudate (9, 9, 4 mm, z = 4.16), left caudate (-11, 5, 9 mm, z = 4.05), right frontal pole / vmPFC (7, 57, -10 mm, z = 3.85), and left frontal pole (-27, 60, -1 mm, z = 3.98). No effect was found for decreased representation of Bayesian updating (blue).

(b) reproduces the effect reported in main Fig. 5, showing overlapping effects for the contrast of contrasts (green) at the same coordinates as in (a).

(c) We also investigated if the effect shown in (a) was rather a reduction of the influence of model-free mechanisms on behavioural belief updating or an increase of the influence of Bayesian updating. This was possible because individual participants' regression weights from the behavioural analysis only shared a relatively small amount of variance (correlation of regression weights across participants r = -0.33, p = 0.11), indicating that the degree of Bayesian updating of a participant was only in part related to the degree of model-free influences across participants. This revealed that increased *RPE* over D_{KL} representation in the dorsal striatum (depicted in red, right caudate 9, 1, 10 mm, z = 4.01; left caudate -13, 3, 11 mm, z = 3.80) as well as bilateral FPC (right 19, 66, 4 mm, z = 3.94; left -21, 64, 8 mm, z = 4.17) was related to decreased influence of model-free learning on belief updating. On the other hand, a dissociable and slightly more posteriorly located region in left FPC (depicted in blue, -27, 52, 7, z = 4.18) covaried with increased influence of Bayesian belief updating in relation to the integration of *RPE* into model-based regions on neural activity. Colourbars indicate z-scores, plots are cluster extent corrected at $p < 0.05$.

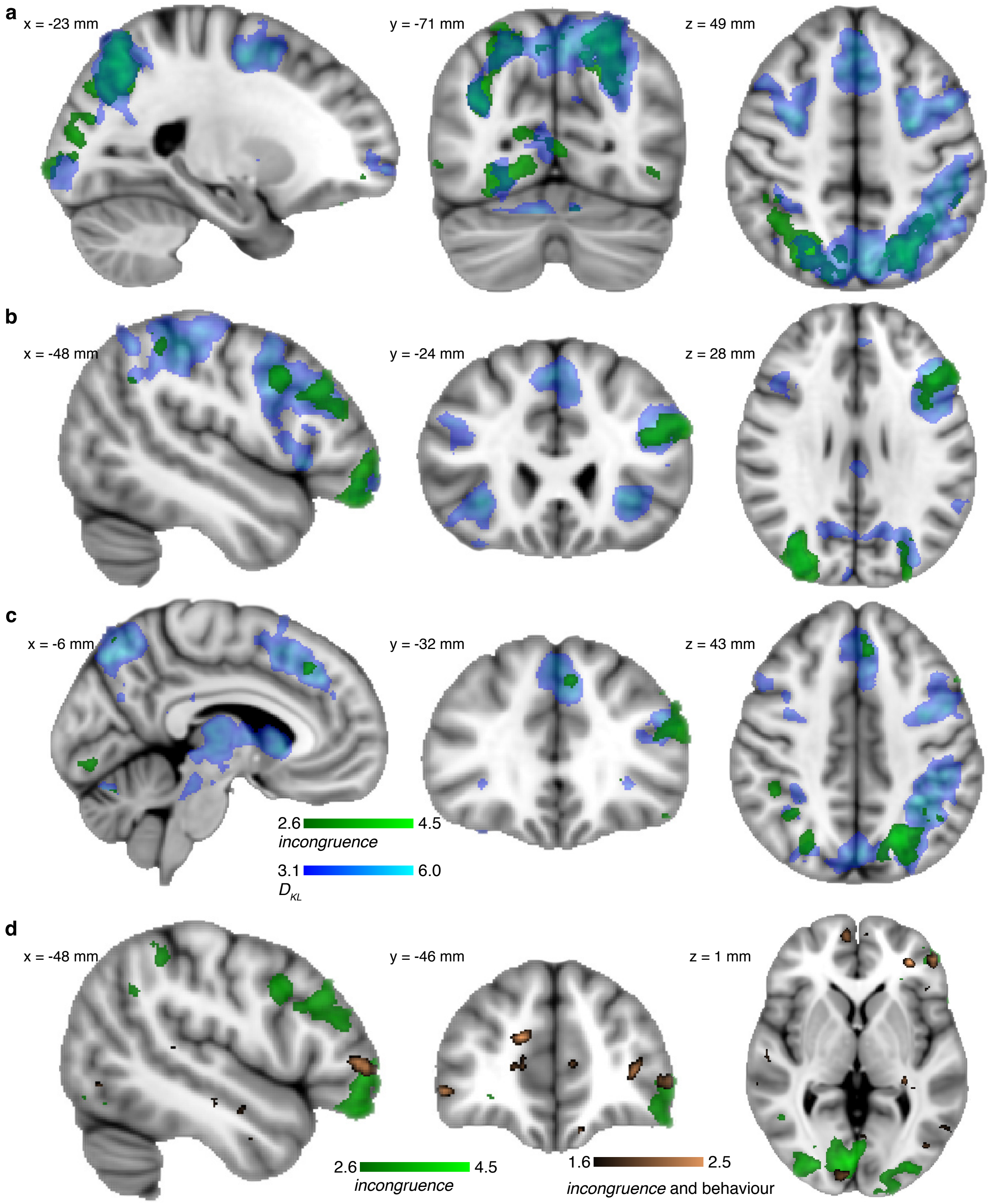

Supplementary Figure 5 |Main effect of incongruence between reward experience and information and overlap with D_{KL}.

We repeated the fMRI analysis with the additional predictor *incongruence* contrasting incongruent and congruent events at outcome presentation. (**a-c**) *incongruence* (green) positively covaried with brain activity in (a) bilateral IPS (peak left -22, -69, 48 mm, z = 4.86, peak right 24, -64, 53 mm, z = 4.70), (**b**) left dlPFC (-42, 21, 26 mm, z = 4.07), and left frontal pole (-46, 44, -2 mm, z = 3.81), all overlapping with main effects seen for D_{KL} (blue). Additionally, at a less

conservative cluster threshold, another cluster in (**c**) pMFC (-5, 30, 42 mm, $z = 3.54$) emerged, again overlapping with a D_{KL} main effect. Note that inclusion of *incongruence* as a regressor in the main GLM did not change results for any of the main effects reported qualitatively.

These results indicate that those brain areas identified in the main analysis as reflecting model-based inference, additionally also reflect mismatch between model-based and model-free updating. A possible explanation is an increased need for cognitive control caused by conflict, which is known to covary with BOLD activity in pMFC as well as dlPFC consistent with detection of the need of increased cognitive control and implementation thereof[8,9].

(**d**) We then also investigated whether individual differences in the degree to which Bayesian updating or model-free learning influenced belief formation covaried with the individual parameter estimates for the *incongruence* regressor in the GLM. The only region that displayed a main effect for *incongruence* as well as a significant covariation with the relative degree of Bayesian updating was the left lateral FPC (-48, 41, 4 mm, $z = 2.37$), additionally overlapping with a main effect for D_{KL}. Thus, the more participants recruited the frontopolar cortex whenever a mismatch between short- and long-term model update occurred, the more participants were able to overcome model-free influences on belief updating.

We also repeated the across-participants analysis from the main manuscript for the parameter estimates of the main effect of *incongruence*. No result survived whole brain FWER correction.

Colourbars indicate z-scores, *incongruence* is thresholded at $p < 0.005$ (uncorrected) (**a-d**), D_{KL} is thresholded at $p < 0.001$, covariation with behavioural influences of model-based and -free learning at $p < 0.05$ (uncorrected) for display purposes (**d**). See also Supplementary Table 1.

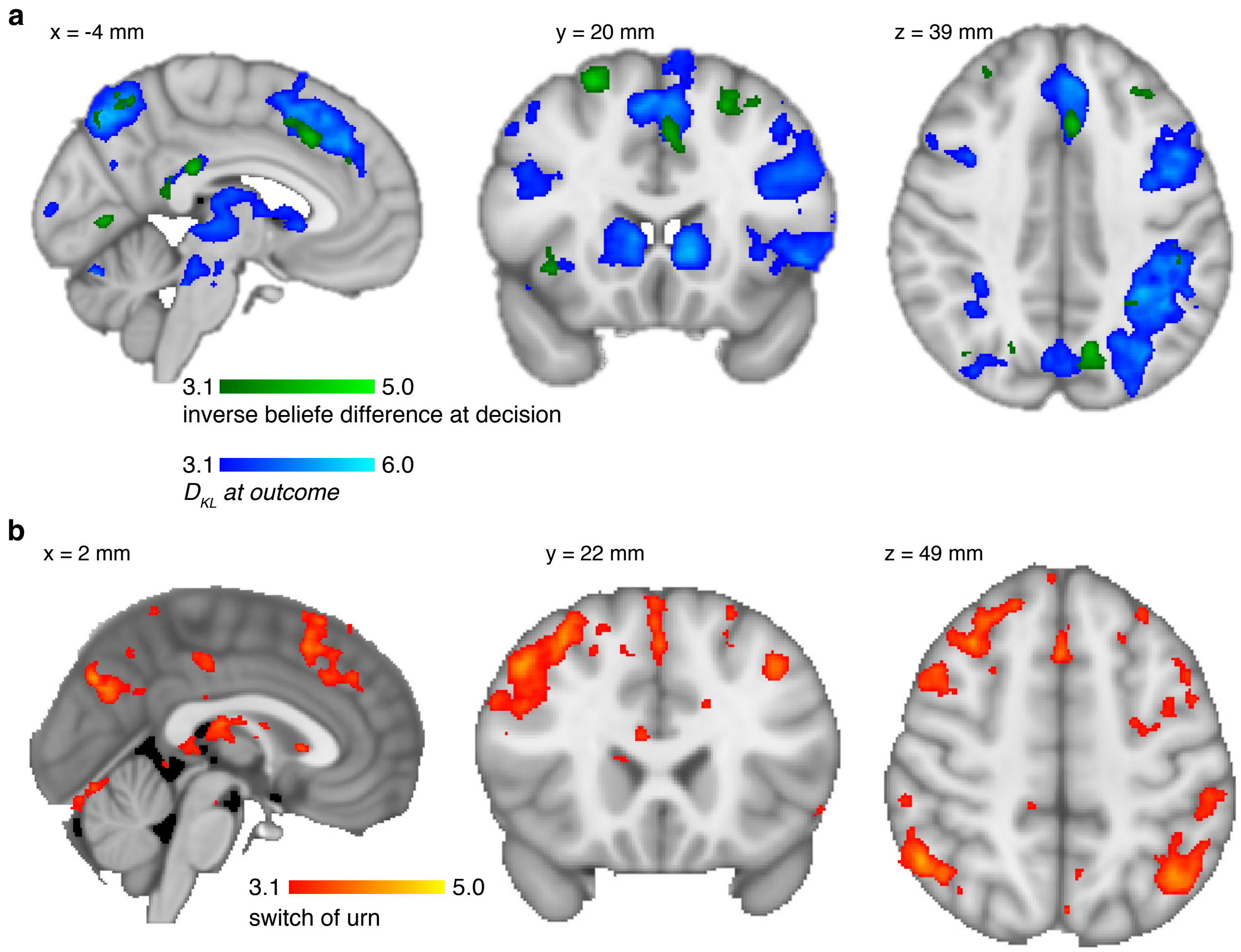

Supplementary Figure 6 | Effects during the decision phase of the experiment.

We furthermore conducted an exploratory analysis of decision related activity in the task for completion. For this analysis, we ran a GLM for which we added a regressor coding the effects of the difference between the chosen and unchosen urns' beliefs, the chosen urn's belief, and if the response was switched or repeated (if a participant chose the same urn as on the previous trial, or not).

(**a**) We found negative effects for belief difference in pMFC (peak -2, 16, 43 mm, z = 3.58), bilateral dlPFC (left -23, 10, 59 mm, z = 4.58; right 22, 13, 62 mm, z = 3.39), IPS (left -9, -62, 56 mm, z = 4.04; right 6, -61, 59 mm, z = 4.23), left FPC (-33, 49, 10 mm, z = 3.89), and PCC (-2, -43, 22 mm, z = 4.49), similar to an inverse value difference signal reported previously[10]. There were no positive believe-difference effects that survived FWER correction. (**b**) We furthermore found significant additional positive switch effects in similar cortical regions (pMFC 1, 10, 49 mm, z = 4.03; dlPFC 26, 35, 47 mm, z = 4.75; left FPC -27, 58, 22 mm, z = 4.6), consistent with a cognitive control network. No negative switch effects were seen following cluster correction.

Note that the timing of the task is not optimized to study decision-related activity, and we therefore urge caution in interpreting these findings. Specifically, because participants had unlimited time to enter their beliefs during the prompt,

they may have already formed decision about upcoming choices at this stage.

Colourbars indicate *z*-scores, all effects displayed are cluster extent corrected.

Supplementary Table 1: List of clusters of activation found for main effects and control analyses.

	n voxels	peak *z*-score	x	y	z
**D_{KL} positive effects**					
(mainly left) IPS, left visual cortex, cerebellum	86475	6.02	-32	-47	43
pMFC, bilateral dlPFC	51696	6.5	-26	-5	52
bilateral caudate	19411	5.94	-9	16	2
right visual cortex	5067	4.2	-29	-94	-11
left FPC	4971	5.07	-35	60	6
right insula	2426	5.59	33	24	-4
brain stem / midbrain	2065	4.28	0	-26	-22
right IPS	1264	3.94	29	-45	45
PCC	863	4.09	0	-28	30
**D_{KL} negative effects**					
right TPJ	5122	4.85	54	-33	28
left insula, frontal operculum	3432	4.73	-37	7	6
vmPFC	3087	4.47	5	61	6
right insula, frontal operculum	2616	4.64	41	-2	-14
right amygdala, parahippocampal cortex	2347	4.69	22	-2	-18
right OFC	1517	5.48	28	34	-12
left TPJ	1230	4.29	-58	-34	18
left OFC	1203	5.57	-29	34	-10
left intracalcarine cortex	798	4.04	-17	-81	8
left amygdala	672	3.93	-23	-2	-18
***RPE* positive effects**					
left visual cortex	17542	6.92	-12	-82	-16
right ventral striatum	7095	5.57	19	11	-5
left ventral striatum	5475	5.1	-18	8	-7
vmPFC, FPC	5292	4.89	-4	61	3
PCC	2129	4.03	-1	-34	35
right precuneus	971	4.09	23	-46	17
anterior cingulate gyrus	756	4	7	37	6

	n voxels	peak *z*-score	x	y	z
***RPE* negative effects**					
right visual cortex (V1, V2, V3)	26944	6.69	14	-95	3
left visual cortex (V1)	2047	4.53	-14	-81	33
**ΔB_t positive effects**					
right occipital pole	5572	5.2	2	-89	8
**ΔB_t negative effects**					
right visual cortex (V2, V3)	5298	5.46	11	-75	-6
***Incongruence* positive effects**					
right IPS	9858	4.7	24	-64	53
right visual cortex (intracalcarine cortex)	8599	4.82	6	-84	2
left IPS	7108	4.86	-22	-69	48
left dlPFC	1569	4.07	-42	21	26
right inferior temporal gyrus	929	4.09	51	-57	-8
left lateral frontal pole	863	3.81	-46	44	-2
***Bayesianness* positive effects**					
aMCC	6934	4.71	-9	37	27
right striatum	4341	4.37	13	13	-9
precentral gyrus	3488	4.25	-35	-25	46
left striatum	2834	4.34	-15	12	9
left insular cortex / OFC	2686	4.71	-36	17	-11
PCC	2440	4.68	-1	-19	33
right insular cortex / OFC	2320	4.39	41	17	-11
angular gyrus, supramarginal gyrus	1515	3.94	-62	-49	29
posterodorsal thalamus, superior colliculus	1295	4.31	-6	-27	-6
left dorsolateral FPC	1283	4.29	-26	35	28
midbrain (VTA, SN, raphe)	1164	4.05	-2	-19	-18
cerebellum	1100	4.57	15	-53	-20
IFG	788	4.21	-53	22	-2

Please note that despite the conservative threshold (cluster $p < 0.05$ and activation $p < 0.001$) used here, cortical clusters sometimes revealed very large activation patterns, that spanned multiple areas. D_{KL}: divergence Kullback-Leibler; *RPE*: reward prediction error; ΔB_t: directed belief updating or signed divergence Kullback-Leibler; pMFC: posterior mesial frontal cortex; dlPFC: dorsolateral prefrontal cortex; FPC: frontopolar cortex; PCC: posterior cingulate cortex; IPS: intraparietal sulcus; vmPFC: ventro-medial prefrontal cortex; TPJ: temporoparietal junction; OFC: orbitofrontal cortex; PCC: posterior cingulate cortex; VTA: ventral tegmental area; SN: substantia nigra; IFG: inferior frontal gyrus.

Supplementary Note 1

A potential confound of the task design is that the belief bar marker that participants moved to indicate their beliefs following each trial, remained in place. Thus, it would be possible to solve the task without forming actual beliefs about long-term valences of the urns, as it would be sufficient to decide in which direction to move the marker and remember its final position to solve the estimation of urns between blocks correctly. This appears especially important, because the dorsal striatum has repeatedly been implicated in decision formation processes, rather than learning[11,12]. Although follow-up surveys did not indicate that participants used the (spatial) position of the bar as the basis for their choices, we conducted a replication study to rule out this possibility and ensure that the neural correlates of belief updating are truly related to inferential learning.

To this end, we introduced the following changes to the task. Firstly, participants were no longer prompted about their beliefs after every trial, yet only after every third trial. They could thus predict whether they would be asked to enter their beliefs, or not. This tests if prompting beliefs changed the neural effects we found. Secondly, the belief prompt was always set back to 0.5, the point of indifference. Thus, participants had to form and maintain beliefs about good and bad urns and could not rely on the spatial information provided by the prompt. This therefore excludes that the task could be solved without an internal representation of long-term valence of the urns.

Supplementary Methods

We measured an additional sample of 21 participants for fMRI analyses, out of which 18 (mean age 23, 13 female) finished the recording session (3 left after the task was completed, but before the structural image could be acquired due to tiredness or strangury). All participants were informed about possible risks of the measurements prior to participation, and all procedures were carried out in accordance with the declaration of Helsinki. The study protocol was approved by the ethics committee of the medical faculty of the Otto-von-Guericke University, Magdeburg (Germany).

All data for the replication was collected on a 3T Siemens Skyra scanner and preprocessing was identical to the original study, with the exception that no field map correction was applied. We again used an isotropic resolution of 3 mm, TR = 2s, TE = 30 ms, and a flip angle of 80° for the functional recording. The mean number of acquired volumes was 1590 (range 1388 to 1999), mean task duration 53 minutes, comparable to the original study. Settings for the T1 MPRAGE were TR = 2320 ms, TE = 2.96 ms, TI = 1200 ms, otherwise identical to the original protocol.

As we could not fit the RL model to the participants' sequences of beliefs, because these were not available on a trialwise basis, we used the best fitting learning rate from the original study (0.06) to derive the *RPE* regressor. We used

the same Bayesian model as in the original study, based on the belief participants entered on every third trial. The GLM was otherwise set up identically, but included an additional regressor that coded if a belief would be prompted after the feedback period, as well as the interaction between this regressor and D_{KL}, RPE_t, and ΔB_t, respectively. Additionally, we ran an analysis in which we coded D_{KL} separately for trials with prompts, and trials without prompts. With this we test the hypothesis that beliefs are only formed when participants know they should be entered afterwards. If the interaction is not significant and main effects for D_{KL} remain significant even when only trials are analysed in which participants know they will not be prompted, this indicates that belief updates and their neural correlates were not confined to prompt trials.

Participants earned on average 118 ± 9 (SE) points, and chose the good urn when one was good and the other one was bad (GB blocks) on 132 ± 3 and the bad one on 108 ± 3 trials (t_{17} = 3.65, p = 0.0002 for difference). Thus, they chose the better urn on slightly fewer trials compared to the original study (mean 21 trials, t_{40} = -3.3, p = 0.002), yet still successfully more often than the bad one.

As in the original study, participants successfully formed beliefs about good and bad urns (Fig. 7a). At the last prompted trial of good urns in GB blocks, they entered a belief of 0.667 ± 0.021 (t-test against indifference (0.5) t_{17} = 8.84, p < 10^{-6}) and on bad ones 0.419 ± 0.019 (t_{17} = -4.36, p < 0.0005). In blocks where both urns were good, this was 0.58 ± 0.028 (t_{17} = 3.11, p = 0.0064) and when both urns were bad this was 0.394 ± 0.027 (t_{17} = -4.04, p = 0.00086).

Thus, participants established robust beliefs about good and bad urns even when they could not rely on the previous position of the belief marker. Given that participants were well able to identify the urns' long-term valence between blocks (Fig. 7a), this indicates that while they were slower to establish beliefs (slightly less choices of the good urns), they still formed robust beliefs about the urns' long-term valences. The difference to the original study is likely explained by the increased difficulty when the belief has to be remembered during trials.

On a neural level, we replicated the striatal dissociation found in the original study (Fig. 7b). We also replicated all major main effects seen for D_{KL} and these overlapped with the D_{KL} effects in the original study even when a whole brain cluster correction and a p threshold < 0.001 was applied. We found significant effects in the left (peak -9, 10, 10 mm, z = 3.72) and right dorsal striatum (7, 15, 2 mm, z = 4.06), left (-35, -50, 43 mm, z = 4.07) IPS, left dlPFC (-35, 11, 33 mm, z = 4.9), pMFC (-4, 25, 43 mm, z = 4.6), and left FPC (-31, 52, 17 mm, z = 3.61). All these effects remained significant when we analysed only trials without a following belief prompt (Fig. 7c).

The effects for *RPE* were somewhat reduced, possibly because of the worse fit possibility of the RL model. We still found overlapping significant effects in the left (Fig. 7d, -14, 17, -6 mm, z = 2.50) and right ventral striatum (13, 19, -8

mm, z = 2.77). Furthermore, we found significant effects overlapping with the original cluster spanning vmPFC (20, 45, -3 mm, z = 2.26) and FPC (-2, 65, 6 mm, z = 2.67).

The only significant interaction effect between belief prompting and D_{KL} or *RPE* was seen in the left IPS (-42, -46, 42 mm, z = 3.3), indicating that D_{KL} was more strongly reflected here when participants knew that they would be prompted to enter their current belief estimate afterwards. Without cluster extent correction, interactions also emerged for the left dlPFC (-45, 21, 31 mm, z = 3.06), pMFC (3, 22, 53 mm, z = 2.9), but not the dorsal striatum or the FPC. In sum, these data indicate that participants updated beliefs mostly independent of whether they would be prompted to enter them in a trial, as is appropriate to solve the task. The replication furthermore suggests that in both tasks, participants formed internal representations of belief states which were updated via inference derived from the payouts and knowledge of the event distributions.

Supplementary References

1. Xie, J. & Padoa-Schioppa, C. Neuronal remapping and circuit persistence in economic decisions. *Nat. Neurosci.* **19,** 855–861 (2016).
2. Padoa-Schioppa, C. Range-Adapting Representation of Economic Value in the Orbitofrontal Cortex. *Journal of Neuroscience* **29,** 14004–14014 (2009).
3. Alexander, W. H. & Brown, J. W. Medial prefrontal cortex as an action-outcome predictor. *Nature Publishing Group* **14,** 1338–1344 (2011).
4. Ullsperger, M., Fischer, A. G., Nigbur, R. & Endrass, T. Neural mechanisms and temporal dynamics of performance monitoring. *Trends in Cognitive Sciences* **18,** 259–267 (2014).
5. Schultz, W. Dopamine reward prediction-error signalling: a two-component response. *Nature Reviews Neuroscience* **17,** 183–195 (2016).
6. Caplin, A. & Dean, M. Axiomatic methods, dopamine and reward prediction error. *Current Opinion in Neurobiology* **18,** 197–202 (2008).
7. Daw, N. D., Gershman, S. J., Ben Seymour, Dayan, P. & Dolan, R. J. Model-Based Influences on Humans' Choices and Striatal Prediction Errors. *Neuron* **69,** 1204–1215 (2011).
8. MacDonald, A. W., Cohen, J. D., Stenger, V. A. & Carter, C. S. Dissociating the Role of the Dorsolateral Prefrontal and Anterior Cingulate Cortex in Cognitive Control. *Science* **288,** 1835–1838 (2000).
9. Ullsperger, M., Danielmeier, C. & Jocham, G. Neurophysiology of Performance Monitoring and Adaptive Behavior. *Physiological Reviews* **94,** 35–79 (2014).
10. Rushworth, M. F., Kolling, N., Sallet, J. & Mars, R. B. Valuation and decision-making in frontal cortex: one or many serial or parallel systems? *Curr. Opin. Neurobiol.* **22,** 946–955 (2012).
11. Atallah, H. E., Lopez-Paniagua, D., Rudy, J. W. & O'reilly, R. C. Separate neural substrates for skill learning and performance in the ventral and dorsal striatum. *Nat. Neurosci.* **10,** 126–131 (2007).
12. Hiebert, N. M. *et al.* Striatum in stimulus–response learning via feedback and in decision making. *Neuroimage* **101,** 448–457 (2014).
